# Disentangling How Landscape Spatial and Temporal Heterogeneity Affects Savanna Birds

**DOI:** 10.1371/journal.pone.0074333

**Published:** 2013-09-16

**Authors:** Bronwyn Price, Clive A. McAlpine, Alex S. Kutt, Doug Ward, Stuart R. Phinn, John A. Ludwig

**Affiliations:** 1 The Swiss Federal Institute for Forest, Snow and Landscape Research WSL, Birmensdorf, Switzerland; 2 The University of Queensland,Centre for Spatial Environmental Research, School of Geography, Planning and Environmental Management, St Lucia, Australia; 3 School of Marine and Tropical Biology, James Cook University, Townsville, Australia; 4 School of Botany, University of Melbourne, Parkville, Australia; 5 Griffith University, Australian Rivers Institute, Nathan campus, Nathan, Australia; 6 CSIRO Ecosystem Sciences, Atherton, Australia; University of Georgia, United States of America

## Abstract

In highly seasonal tropical environments, temporal changes in habitat and resources are a significant determinant of the spatial distribution of species. This study disentangles the effects of spatial and mid to long-term temporal heterogeneity in habitat on the diversity and abundance of savanna birds by testing four competing conceptual models of varying complexity. Focussing on sites in northeast Australia over a 20 year time period, we used ground cover and foliage projected cover surfaces derived from a time series of Landsat Thematic Mapper imagery, rainfall data and site-level vegetation surveys to derive measures of habitat structure at local (1–100 ha) and landscape (100–1000s ha) scales. We used generalised linear models and an information theoretic approach to test the independent effects of spatial and temporal influences on savanna bird diversity and the abundance of eight species with different life-history behaviours. Of four competing models defining influences on assemblages of savanna birds, the most parsimonious included temporal and spatial variability in vegetation cover and site-scale vegetation structure, suggesting savanna bird species respond to spatial and temporal habitat heterogeneity at both the broader landscape scale and at the fine-scale. The relative weight, strength and direction of the explanatory variables changed with each of the eight species, reflecting their different ecology and behavioural traits. This study demonstrates that variations in the spatial pattern of savanna vegetation over periods of 10 to 20 years at the local and landscape scale strongly affect bird diversity and abundance. Thus, it is essential to monitor and manage both spatial and temporal variability in avian habitat to achieve long-term biodiversity outcomes.

## Introduction

Globally, savanna ecosystems are an important reservoir of biodiversity, but are undergoing rapid changes due to increased land use pressures including clearing, grazing and changes in fire regimes [1]–[3]. In tropical savanna environments, annual and inter-annual variability in rainfall can have a dramatic impact on ecosystems and their biota [4]-[6]. However, very few studies have examined how temporal variation in habitat and resources at local to landscape scales (1–1000’s ha), relevant to land management, influences species’ distribution and abundance [7], [8]. Difficulties in obtaining historical landscape data results in few studies explicitly considering temporal dynamics of habitat [9], yet it is recognised that temporal habitat dynamics are likely to have significant impact on fauna populations [9], [10].

Seasonal and inter-annual variability in habitat attributes such as cover and resource availability are important components of species’ habitat relationships, and can have an important influence on species’ distribution patterns according to their mobility and ability to utilize changing habitat resources [7], [11]. However, while multi-temporal analysis has been widely applied to quantify landscape change [12], [13], few studies have quantified the relative importance of temporal heterogeneity in habitat attributes on fauna distribution and abundance.

Wildlife respond to temporal variability in habitat attributes in a variety of ways including seasonal migration, nomadic dispersal movement, and shifting local patterns of habitat utilization and population dynamics [14]–[16]. In tropical savannas, many bird species track resources such as nectar from flowering trees [17] whose phenology may be controlled by rainfall patterns. Similarly, regional migration patterns in birds can be controlled by inter-annual and seasonal weather patterns [18], which in turn are driven by annual to decadal La Niña and El Niño climatic patterns [19].

In addition to climatic variability, land use disturbances (e.g. fire, clearing and grazing) can alter vegetation structure and dynamics, particularly at local (1–100ha) to landscape (100–1000s ha) scales and at 10–20 year time scales, with important consequences for the composition and abundance of woodland birds [20].

The capacity now exists to measure temporal and spatial heterogeneity in vegetation cover using historical archives of satellite imagery at moderately high spatial resolutions, i.e. freely available archival annual Landsat Thematic Mapper imagery with a 30 m resolution over a 18 year time span [11], [21]. Temporal changes in herbaceous vegetation cover can be inferred from observed climatic data over the medium to long term. However, multi-temporal remotely sensed imagery provides a more direct and accurate measure of both spatial and temporal heterogeneity in vegetation cover, which is influenced by climate variability and also driven by land management practices at local and landscape scales. Remote sensing imagery is economically attractive and available at appropriate spatial and temporal scales for characterising the temporal dynamism of savanna environments and impacts for biodiversity.

There is a relatively good understanding of local-scale (1–10 ha) relationships between savanna bird abundance and diversity, and habitat structure, composition and disturbance regimes [22]. However, the interactive effects of spatial and temporal landscape heterogeneity on fauna are often poorly understood (sensu [7]). In the tropical savannas of northeast Australia, Ward and Kutt [11] demonstrated that a precipitation deficit index and remotely-sensed ground cover measured at the site-scale (4 ha) were significant predictors of woodland bird diversity. However, their study did not account for spatial or temporal heterogeneity at the landscape-scale, where anthropogenic disturbances (e.g., clearing, grazing), on top of climatic factors, may have an important influence on vegetation cover and dynamics. There are several unresolved questions regarding how the diversity, distribution and abundance of savanna birds are influenced by broad-scale climatically driven temporal heterogeneity in vegetation cover, and the relative importance of temporal and spatial heterogeneity at the landscape scale driven by management practice.

Rapid global change is the most compelling issue for conservation sciences at present [23], and understanding the relative influence of temporal and spatial factors and land management practice on species’ distributions is a significant component of mitigation and management [7]. Although species’ distributions are driven by multi-scale factors, land management for conservation in savannas often considers only the local scale [24]. Land management practice can have a strong influence on vegetation cover particularly at the local to landscape scale (1–1000’s ha) [20]. We therefore hypothesise that local scale temporal and spatial habitat variability may be of equal or greater importance for bird diversity and abundance as landscape to regional scale heterogeneity, which can be considered to be largely driven by rainfall. However, the relative importance of spatial heterogeneity, temporal heterogeneity and scale is likely to vary between individual species given their differing behavioural traits and habitat preferences.

This study addresses the following questions for explaining spatial variation in bird diversity and abundance in tropical savanna landscapes: i) how important is temporal heterogeneity in vegetation cover relative to spatial heterogeneity? ii) how influential is local to landscape-scale heterogeneity in vegetation cover compared to regional-scale rainfall-driven variation in vegetation cover?

## Methods

### 2.1 Conceptual Model

**Figure 1:**
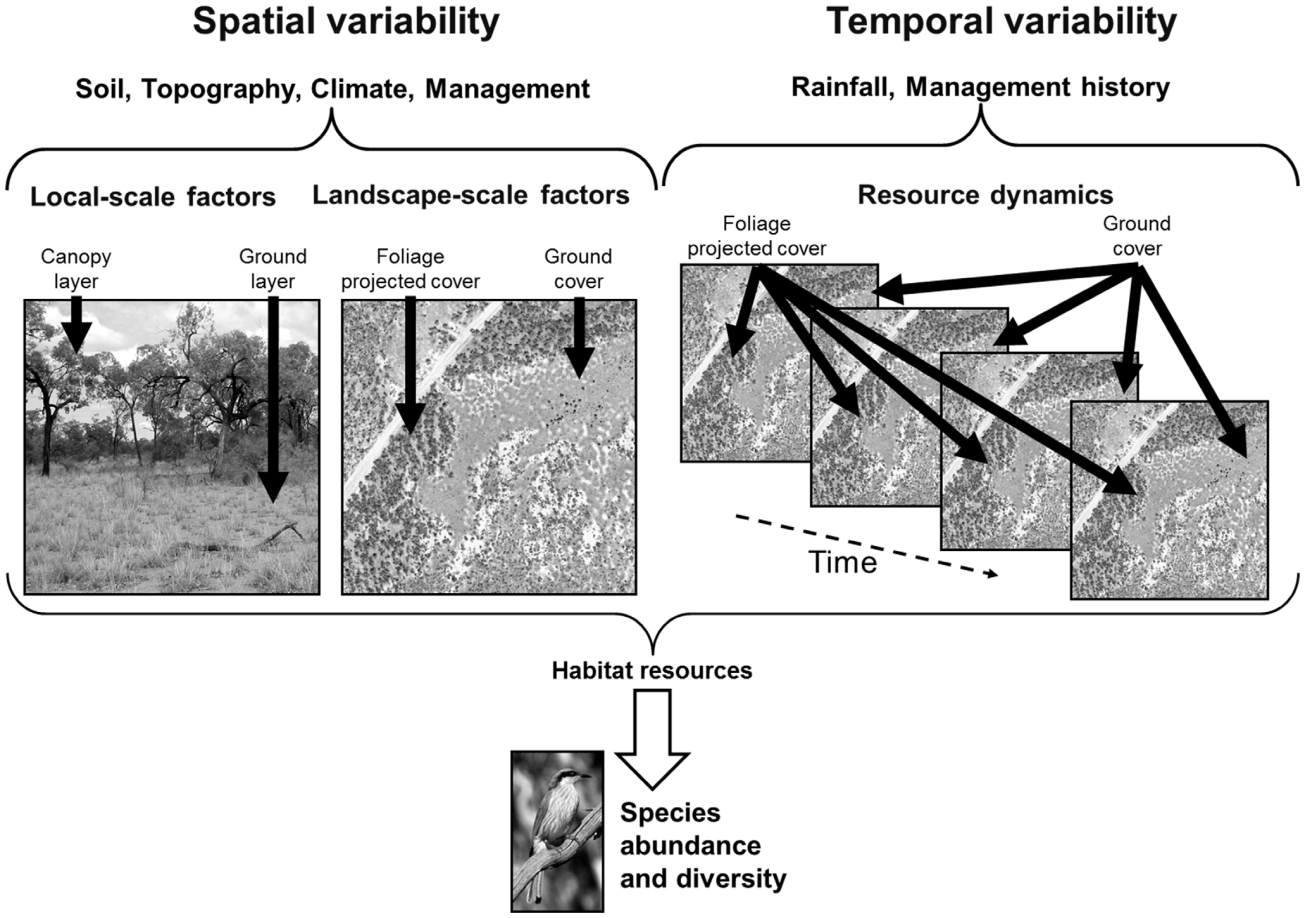
Conceptual model of how species’ abundance and diversity are influenced by the spatial and temporal variability in habitat (woody and herbaceous vegetation elements) structure for a single savanna ecosystem.

We developed a conceptual model of how spatial and temporal factors may influence savanna bird assemblages at different scales, and from this identified four competing hypotheses to disentangle effects of spatial and long-term temporal heterogeneity in habitat, and rainfall-driven (at landscape to regional scales) and management-driven (at local to landscape scales) heterogeneity (Figure 1). In this conceptual model, habitat is species’ specific and forage or shelter resources are linked to presence of vegetation elements that are important for an individual species (e.g. tree canopy and grass cover) (e.g., [25]). We conceptualise that spatial heterogeneity in habitat structure (tree and ground-layer herbaceous vegetation cover) in savanna landscapes is driven by spatial variability in soil, topography, climate and land management practices such as clearing and fire regimes; while temporal heterogeneity is influenced by preceding rainfall patterns at the regional to landscape scale and over temporal scales up to 18 years, and by fire and grazing management at local to landscape scales and also over temporal scales up to 18 years (Figure 1).

We postulate four competing models:

1. Local habitat model: local-scale (1–100ha) habitat structure explains diversity and abundance of savanna birds. Spatial and long term temporal heterogeneity at the landscape-scale is of less importance.
2. Spatial heterogeneity model: the spatial landscape context is important for savanna birds. Consideration of variables describing heterogeneity across the landscape allows better prediction of savanna bird diversity and abundance.
3. Temporal rainfall variability model: rainfall variability is the key driver of temporal variability in habitat attributes for savanna birds. Broad-scale temporal variability in rainfall in conjunction with local-scale vegetation structure are important predictors of savanna bird diversity and abundance.
4. Temporal and spatial heterogeneity model: spatial and temporal heterogeneity in woody and herbaceous vegetation cover at local and landscape scales are important predictors of savanna bird diversity and abundance. Annual temporal heterogeneity in vegetation cover across the landscape reflects the impact of land management as well as broader scale climatic gradients.

These four models were then used as a basis for constructing a set of alternative statistical models for testing the relative influence of spatial and temporal heterogeneity in habitat attributes at local and landscape scales on savanna bird species richness and abundance.

### 2.2 Study Area

**Figure 2:**
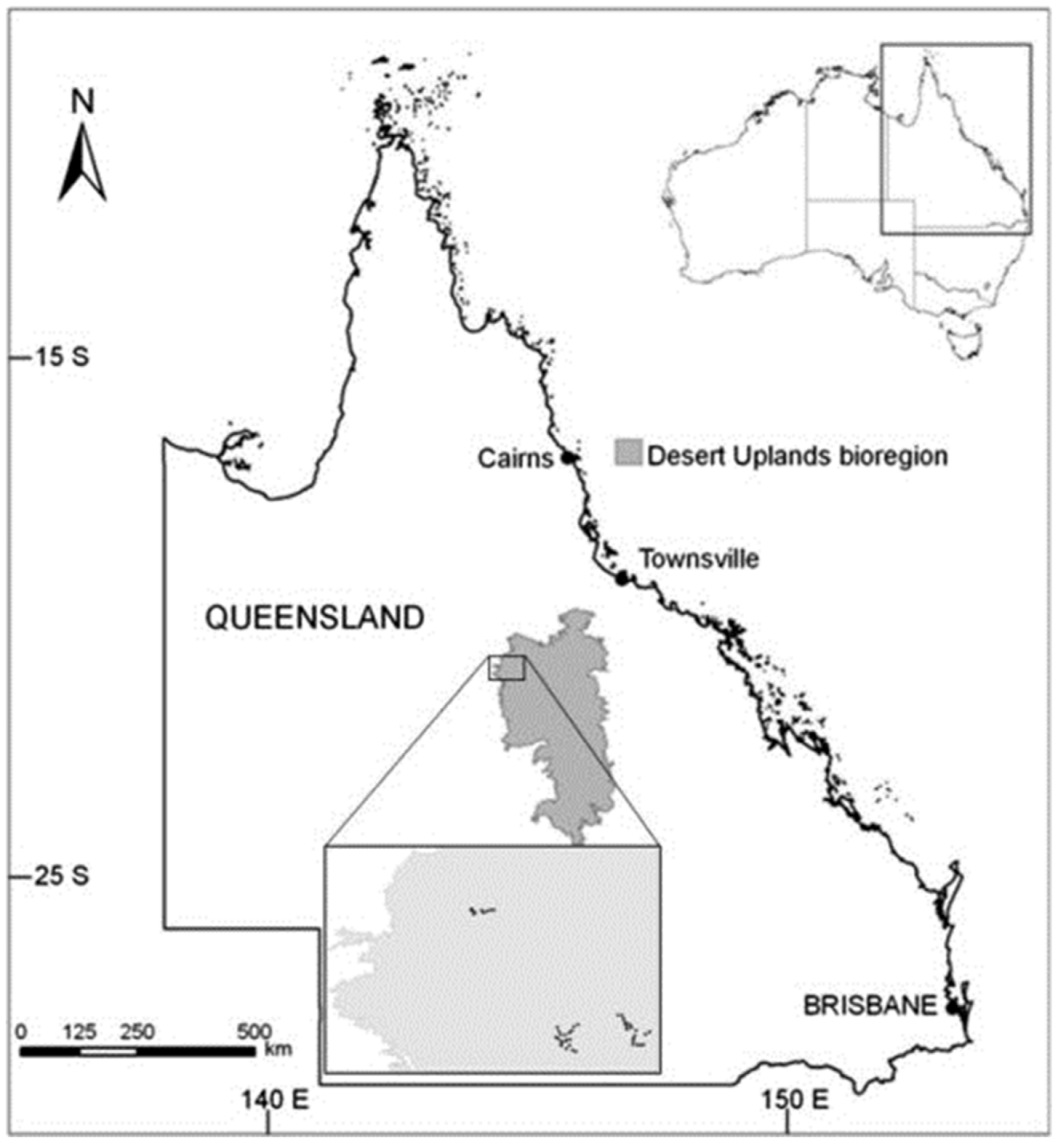
Location of case study area with sites (inset).

The study focused on the Desert Uplands bioregion of Queensland, Australia (Figure 2), which has a semi-arid climate with a mean annual rainfall in the range of 350–600 mm. Vegetation consists predominantly of Acacia and Eucalyptus open woodlands (height <15 m), ephemeral lakes and grasslands [26]. Open woodlands occurring on sandy soils occupy ∼85% of the region. Beef cattle grazing is the major form of primary production and was established in the mid-nineteenth century [27]. Much of the region is considered of low potential for pastoralism due to relatively low rainfall, poor soils and low general palatability of the vegetation for stock [28]. The grazing industry is based largely on the extensive use of unimproved natural rangelands and most properties in the region are >20,000 ha in size. The soils are considered of low to moderate fertility, phosphorous deficient and grazing is restricted to approximately 30% utilisation of the available area in this vegetation type. Vegetation cover is also influenced at local to landscape scales by fire (both natural and anthropogenic) and clearing [29].

Sites were located in a single regional ecosystem type, (10.3.9, [26]), defined as extensive open-woodlands of silver-leafed ironbark (*Eucalyptus whitei*), characteristic of low-fertility eucalypt savannas of northeast Australia. The location of the sites in a same ecosystem type ensured variation in the observed bird patterns was not influenced by spatial differences in vegetation communities. All surveys were conducted on leasehold land and permission to access and survey these properties were obtained by the landholders, namely the Bode Families (Woura Park, Timaru) and Haydon Family (Penrice).

### 2.3 Bird Surveys

Fifty 1-ha sites were surveyed in May-June 2004 and resurveyed in March-April 2005 and July-August 2006. Within each 1-ha site, eight five-ten minute diurnal bird counts were conducted over a four day period, and visual and aural observations were recorded. Each count represented the entire 1-ha area. At least two bird counts of each 1-ha plot were made per day at each site; one count in the morning between dawn and three hours after dawn, and the other a minimum of three hours after this period and before dusk. Studies of extensive bird survey data (over 500 sites sampled over 6 years) for these open, largely homogenous tropical savannas have demonstrated that repeated sampling of 1 ha areas, over multiple days and at different times of the day is the most appropriate method to count bird assemblages often dispersed across the landscape, and that distance sampling methods are not required [30].

Data used in the analyses were the total summed relative abundance of all the eight counts in each site for each year. All sites were located a minimum of 500 m from watering points to standardize the impact of grazing pressure across sites, and as far as practical from fence lines and roads. Sites were separated by a minimum of 1 km to avoid spatial dependence and all sites are located within a discrete unit of the mapped vegetation polygon (i.e. away from edges). Birds were recorded for all surveys at all sites, with each site having more than five species in total over the course of that season’s survey.

To take into account both species richness and abundance, we calculated Shannon diversity index (*H*) [31], which also provides a standard measure of diversity not biased toward common or rare species as we were not interested in those species *per se* [32]. We also selected eight bird species with varying habitat preferences for testing the alternative models: crested bellbird *Oreoica gutturalis*, double-barred finch *Taeniopygia bichenovii,* grey-crowned babbler *Pomatostomus temporalis*, grey shrike thrush *Colluricincla harmonica,* singing honeyeater *Lichenostomus virescens*, weebill *Smicrornis brevirostris,* yellow-throated miner *Manorina flavigula* and zebra finch *Taeniopygia guttata*. These species represent the range of foraging and nesting guilds typical of woodland birds in the study area (see Table 1 for summary of behavioural traits). These eight species utilise the full scope of the vertical vegetation structure, and were also species for which we had sufficient data for statistical modelling.

**Table 1:** Summary of behaviour of individual bird species used as response variables [33]–[35].

Common name	Scientific name	Guild	Foraging	Nesting	Migration
Crested bellbird	*Oreoica gutturalis*	Ground, understorey insectivore	Ground level, low shrubs, trees. Forages individually.	Broken tree branches (spouts), stump hollows, tree crotch, dense undergrowth (usually <3 m from ground)	Resident to sedentary
Double-barred finch	*Taeniopygia bichenovii*	Granivore	Ground level, directly fromseed heads on grass tussocks.Forages in small tolarge groups	Shrubs, small trees or grasstussocks <3 m from the ground	Resident to sedentary
Grey-crowned babbler	*Pomatostomus temporalis*	Ground and foliage insectivore, omnivore	All strata: canopy, trunks,branches, low shrubs andground. Forages insmall family groups.	Tree forks or dense foliage ofshrubs and trees from 2–15 mabove ground	Resident to sedentary
Grey shrike thrush	*Colluricincla harmonica*	Omnivore	Ground level, limbs, trunks oftrees. Forages individually	Ground level, dense shrubs orgrass	Resident to sedentary
Singing honeyeater	*Lichenostomus virescens*	Foliage insectivore, nectarivore	Shrub, mid-strata and canopy trees. Forages individually.	Dense shrubs, saplings, tree branches 2–3 m above theground level	Resident to sedentary
Weebill	*Smicrornis brevirostris*	Foliage insectivore	Canopy, sub-canopy trees, shrub layer. Forages in small groups.	Leafy foliage in trees or shrubs,1–10 m above the ground	Resident to sedentary
Yellow-throated miner	*Manorina flavigula*	Foliage insectivore, nectarivore	Canopy, sub-canopy. Also low trees and shrubs. Forages individually or in small groups.	Dense shrubs, saplings, tree branches 3–5 m above theground level	Resident to sedentary
Zebra finch	*Taeniopygia guttata*	Granivore	Ground level, directly from seed heads on grass tussocks. Forages in small to large groups.	Shrubs, small trees or grasstussocks <3 m from the ground	Sedentary to nomadic (based on rainfall cycles)

This study was carried out in strict accordance with the Queensland Scientific Purposes Permit number WISP11870412 issued under the Nature Conservation (Administration) Regulation 2006 and the (Australian) Commonwealth Scientific and Industrial Research Organisation Ecosystems Sciences Animal Ethic Committee Animal (Permit Number: 2AR 09–09). All surveys were by observation and no trapping or handling of birds was undertaken in this study.

### 2.4 Local-Scale Habitat Variables

At the local-scale, habitat variables measuring vegetation structure and composition were recorded for each site. Basal area was measured from two diagonal corners of the 50 m by 50 m plot for live and dead trees. Mean basal area for each tree and size class and total live and dead basal area was calculated. Horizontal foliage projective cover was visually estimated for six height classes (0–0.5 m representing ground vegetation). Measures of percentage cover of bare earth, rock, litter, grass, sedges, herbs and forbs, and logs (>5 cm) were derived from 20 0.5– m^2^ quadrats in a regular grid within each 50 m by 50 m plot. From this, we calculated the mean cover score for the full plot. Total tree and shrub frequency was calculated as the number of 0.5– m^2^ quadrats over which a tree or shrub was present.

### 2.5 Landscape Scale and Time Series Data

Spatial and temporal variability in rainfall were derived from climatic data spatially interpolated from weather stations to 5-km grids on a daily time step and averaged to monthly surfaces for the period 1988 to 2006 [36]. The mean and standard deviation of monthly rainfall records were calculated for three time intervals comprising short term (5 years), intermediate term (10 years) and long term (18 years – length of series). Local seasonal precipitation variability relative to inter-annual mean precipitation was quantified using Foley’s precipitation deficit index [37] for a 3 year lag period. Foley’s precipitation deficit index was measured for the month of the bird surveys and is rainfall deficit standardized for mean annual precipitation over a specified lag period. Fensham and Holman [38] found that 3 years is a significant lag period for a precipitation deficit to influence tree dynamics in Australia’s tropical savannas.

An annual time-series of foliage projected cover and ground cover was derived from Landsat TM satellite imagery for the period 1988 to 2006. Foliage projected cover (FPC) was estimated by an empirical relationship between basal area, reflectance data and FPC, derived through regression analysis [39]. A ground cover index of total ground cover was derived from the same Landsat TM images using a multiple regression approach. The ground cover index was masked for areas with greater than 20% FPC, where denser canopy coverage makes estimation of ground cover difficult [40].

We calculated statistics for the ground cover and FPC surfaces using 5-km grids, which matched the resolution of the rainfall grid. Bird survey sites were overlaid on the grids with the number of bird survey sites located in each grid cell varying between four and eight. To measure landscape spatial heterogeneity for the dates for which we have bird survey data (2004, 2005 and 2006), we calculated a spatial mean and coefficient of variation of ground cover and FPC for each 5-km grid square.

Temporal variability in habitat for each site was measured from time series statistics for the mean value of FPC and ground cover in a 200 m circular buffer around each site (similar size to 1-ha field survey sites). Exploratory analysis of the FPC and ground cover time series data revealed approximately cyclical patterns of 8–10 years, particularly apparent in the ground cover data, suggesting significant temporal autocorrelation in vegetation cover. Autoregressive modelling using the “arima” package in R (version 2.8.0; http://www.r-project.org) revealed significant positive temporal autocorrelation in FPC and ground cover at a 1 year lag for all sites as well as autocorrelation up to a 5 year lag, which was not always significant. Based on the autoregressive analysis, mean and variance time series statistics were calculated for ground cover and FPC over the short term (5 year autocorrelation lag) and long term (full time series). The autoregressive coefficients for 1 year and 5 year lags were also included in the explanatory variables as measures of temporal autocorrelation.

### 2.6 Statistical Modelling

We used a multivariate generalized linear modelling approach to investigate the explanatory power of site scale variability, landscape scale variability and temporal variability on the diversity and relative abundance of woodland bird species. The response variables were Shannon diversity of birds and the relative abundance of the eight individual woodland bird species. Based on the conceptual models, we constructed a set of four alternative statistical models, using combinations of the suite of explanatory variables for each response variable (Table 2). Model 1, the local-habitat model, acts as the null-model.

**Table 2:** Subset of explanatory variables used in final models where fpc = foliage projected cover, gc = ground cover, cov = coefficient of variation, long term = 20 years and short term = 5 years.

Local habitat (model 1)	Spatial heterogeneity(model 2)	Temporal rainfall variability(model 3)	Temporal and spatial heterogeneity(model 4)
% bare ground	current year mean fpc (5 km)	foley's index	gc autocorrelation 1 year lag
% forb over	current year mean gc (5 km)	long term mean rainfall	gc autocorrelation 5 year lag
% litter cover	current year cov fpc (5 km)	long term variance in rainfall	fpc autocorrelation 1 year lag
% grass cover	current year cov gc (5 km)	short term variance in rainfall	fpc autocorrelation 5 year lag
fpc >10 m	Four site level variables(as for model 1)	current year rainfall	long term mean fpc
fpc <10 m		Four site level variables(as for model 1)	long term mean gc
total basal area			long term cov gc
mean tree density (site)			Four site level variables (as for model 1)
variance in tree density (site)			

High co-linearity among explanatory variables can lead to high standard errors and difficulties in interpreting parameter estimates in generalized linear models [41]. Therefore, we did not include pairs of explanatory variables with Spearman pair-wise correlation coefficients >0.5 in the same model. All models were fitted using R. The Gaussian distribution was used to model Shannon’s Diversity Index. However, examination of the relative abundance data revealed that the data was zero-inflated for most species, resulting in model over-dispersion [42]. We subsequently applied a negative binomial model using the “glm.nb” function in the MASS package of R [43].

We reduced the local-scale habitat variables to a subset of four variables that were not significantly correlated per species based on univariate generalized linear modelling (family = negative binomial), ranking according to Akaike’s Information Criterion (AIC) values [44], and ecological interpretation of correlated variables.

The time series statistics that measure similar characteristics, but at different temporal extents (e.g. mean FPC over the short term and long term), were generally found to be highly correlated. Univariate modelling and variable ranking showed that long-term statistics were always ranked higher than the short-term statistics, thus we excluded the short-term statistics from our models. The long-term mean and variance values for FPC were highly correlated with long-term mean and variance values for ground cover, respectively. Examination of the standard deviation in time series of FPC revealed relatively low temporal variability in FPC (5–8%) across all sites. Therefore temporal variance in FPC was not included in the models. The inclusion of either long term mean ground cover or long-term mean FPC in the final models varied among response variables depending on AIC ranking in the univariate generalized linear models. The final set of explanatory variables for each conceptual model is outlined in Table 2.

To determine a final model for each conceptual model and each response variable, we modelled all combinations of the subset of parameters and selected the set of parameters with the best fit under that conceptual model (Table 3). We ranked the models by their AIC values, determining the highest ranking set of parameters for each conceptual model. We then compared conceptual models by highest ranking AIC and calculated the Akaike weight for each conceptual model for each response variable [44]. Using AIC as a method of model selection also decreases likelihood of over-fitting of models as the AIC value penalises against adding more parameters [45]. This also limits favouring of a more complex model purely due to the inclusion of more variables. Akaike weights represent the relative likelihood of a model, given the data and the full set of candidate models [45]. We conducted a comparison of the support for the best approximating model by determining the weight of evidence (as measured by the Akaike weight) in favour of Model *i* being the best model compared to the alternative conceptual models [45]. Using the evidence ratios method [45], we determined which model, if any, was dominant for each response variable.

**Table 3:** By species, final set of parameters with the best fit under each conceptual model.

	Local habitat model	Spatial heterogeneity model	Temporal rainfall variability model	Temporal and spatial heterogeneity model
Shannon diversity birds	% grass cover	% grass cover	% grass cover	% grass cover
	% litter cover	% litter cover	variance in tree density (site)	variance in tree density (site)
	fpc >10m	fpc >10 m	foley's index	current year mean gc (5 km)
	variance in tree density (site)	variance in tree density (site)	long term mean rainfall	fpc autocorrelation 5 year lag
		current year mean gc (5 km)		
Crested Bellbird	fpc >10 m	fpc >10m	fpc >10m	fpc >10m
	% grass cover	% grass cover	% grass cover	mean tree density (site)
	mean tree density (site)	mean tree density (site)	% litter cover	% litter cover
	% litter cover	% litter cover	long term mean rainfall	current year mean fpc (5 km)
		current year mean gc (5 km)		long term cov gc
		current year mean fpc (5 km)		
		current year cov fpc (5 km)		
Double barred finch	total basal area	% grass cover	variance in tree density (site)	% grass cover
	% grass cover	variance in tree density (site)	% grass cover	variance in tree density (site)
	variance in tree density (site)	current year mean gc (5 km)	long term mean rainfall	gc autocorrelation 5 year lag
	% grass cover	current year mean fpc (5 km)		fpc autocorrelation 1 year lag
		current year cov fpc (5 km)		
Grey crowned babbler	% bare ground	% bare ground	% bare ground	variance in tree density (site)
	% grass cover	% grass cover	variance in tree density (site)	fpc autocorrelation 5 year lag
	variance in tree density (site)	variance in tree density (site)	% forb cover	gc autocorrelation 5 year lag
	% forb cover	% forb cover	foley's index	
		current year cov gc (5 km)	long term mean rainfall	
		current year cov fpc (5 km)		
		current year mean gc (5 km)		
Grey shrike thrush	fpc <10 m	fpc <10 m	mean tree density (site)	mean tree density (site)
	% litter cover	% litter cover	foley's index	current year mean gc (5 km)
	mean tree density (site)	mean tree density (site)	long term mean rainfall	gc autocorrelation 5 year lag
	fpc >10 m	fpc >10 m	short term variance in rainfall	
		current year mean gc (5 km)		
		current year cov gc (5km)		
Singing honeyeater	% grass cover	% grass cover	variance in tree density (site)	% grass cover
	variance in tree density (site)	variance in tree density (site)	% forb cover	variance in tree density (site)
	fpc >10 m	current year cov gc (5 km)	short term variance in rainfall	fpc >10 m
	% forb cover	fpc >10 m		fpc autocorrelation 5 year lag
		% forb cover		current year mean gc (5 km)
		current year mean gc (5 km)		current year cov fpc (5 km)
		current year cov fpc (5 km)		fpc autocorrelation 1 year lag
				long term mean fpc
				long term cov gc
Weebill	fpc >10 m	fpc >10 m	mean tree density (site	mean tree density (site)
	% grass coverFPC	% grass coverFPC	long term mean rainfall	gc autocorrelation 5 year lag
	mean tree density (site)	mean tree density (site)		fpc autocorrelation 5 year lag
	% grass cover	% grass cover		long term cov gc
		current year mean fpc (5 km)		
		current year mean gc (5 km)		
		current year cov fpc (5 km)		
Yellow throated miner	fpc >10 m	fpc >10 m	variance in tree density (site)	fpc >10Vm
	total basal area	total basal area	foley's index	current year mean gc (5km)
	variance in tree density (site)	variance in tree density (site)		fpc autocorrelation 5 year lag
	% grass cover	% grass cover		long term mean gc
		current year cov fpc (5 km)		
		current year cov gc (5 km)		
		current year mean gc (5 km)		
Zebra finch	% bare ground	% bare ground	total basal area	total basal area
	total basal area	total basal area	long term mean rainfall	long term mean fpc
	% forb cover	current year mean fpc (5 km)	short term variance in rainfall	gc autocorrelation 1 year lag
		% forb cover		fpc autocorrelation 1 year lag
		current year mean gc (5 km)		gc autocorrelation 5 year lag
		current year cov fpc (5 km)		long term cov gc

We calculated the model averaged parameter estimate and associated unconditional standard error for each explanatory variable in the dominant model for each species. The model-averaged parameter estimate was calculated by summing the value of the parameter estimate multiplied by the Akaike weight (*w_i_*) from all model combinations where the variable occurred [45]. To compare the independent effect of site-scale, landscape-scale and temporal variables, we calculated the independent effect size of explanatory variables using hierarchical partitioning analysis within the hier.part package in R [46]. Hierarchical partitioning analysis separates the percentage independent and joint contribution of each variable relative to the total explanatory power of the model [47].

To test for goodness-of-fit of the best approximating models for each species, we used a graphical method whereby the standardised residuals were plotted against the half-normal scores and overlaid with a simulated envelope. The model was considered a reasonable fit if the observed residuals followed an approximate straight line and fell within the 95% confidence envelope [48]. Using R, we simulated 19 samples of *n* observations using the fitted model as if it were a true model. The minimum and maximum values of the *n* sets of order statistics provided the simulated envelope [49]. The resultant half-normal plots were used to test the fit of the best approximating generalized linear models. The half-normal plots revealed that all of the best approximating generalized linear models had a good fit.

## Results

The bird surveys revealed considerable spatial and temporal variability across sites in abundance and diversity of species across the five years of surveys. The direction and degree of changes in diversity and diversity and individual species presence and abundances were also variable at local, landscape and regional scales, suggesting responses to local land management practice and regional scale climatic variability are species specific.

Models including variables measuring spatial heterogeneity in ground cover and foliage projected cover performed significantly better for woodland bird species diversity and abundance of individual species than those including temporal variability in rainfall but assuming spatial homogeneity across 5-km grid cells. For bird diversity, of the four models, the temporal and spatial heterogeneity model (model 4) performed the strongest based on Akaike weights (Table 4) and for abundances of the eight species, except for the weebill, there was a significant weight of evidence in favour of model 4 being the best model.

**Table 4:** Ranking order of model performance for each response variable according to Akaike weight (in brackets).

	Local habitat (model 1)	Spatial heterogeneity(model 2)	Temporal rainfall variability (model 3)	Temporal and spatialheterogeneity (model 4)
Shannon diversity birds	4 (0.00001)	2 (0.00045)	3 (0.00001)	1 (0.99953)
Crested Bellbird	4 (0.00001)	2 (0.00015)	3 (0.00003)	1 (0.99980)
Double barred finch	3 (0.00000)	3 (0.00000)	2 (0.00001)	1 (0.99999)
Grey crowned babbler	4 (0.00000)	3 (0.00000)	2 (0.00049)	1 (0.99951)
Grey shrike thrush	4 (0.00058)	2 (0.00685)	3 (0.00669)	1 (0.98587)
Singing honeyeater	4 (0.00000)	3 (0.00000)	2 (0.00000)	1 (1.00000)
Weebill	4 (0.20525)	2 (0.25672)	3 (0.20525)	1 (0.33278)
Yellow throated miner	4 (0.00000)	3 (0.00000)	2 (0.00000)	1 (1.00000)
Zebra finch	4 (0.00000)	3 (0.00000)	2 (0.00000)	1 (1.00000)

**Figure 3:**
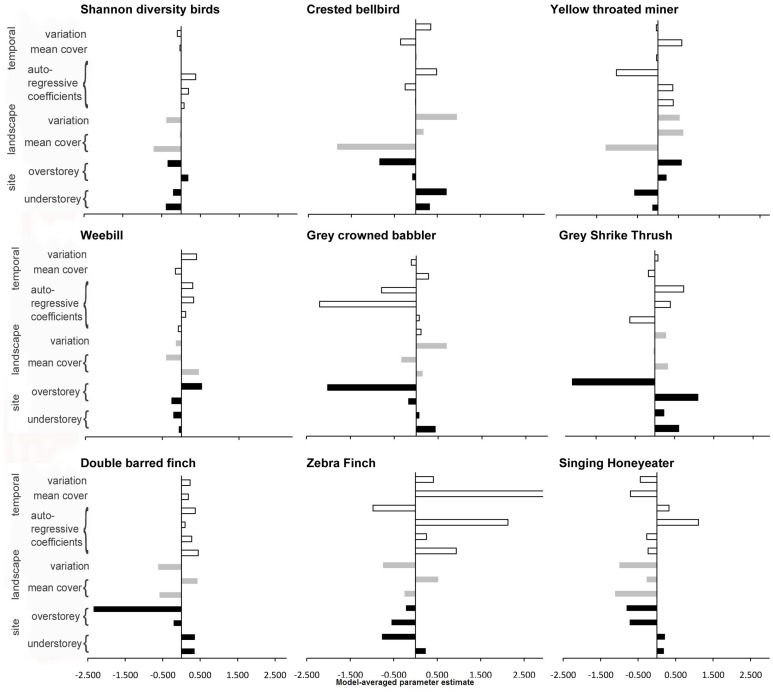
Model averaged parameter estimate of explanatory variables for each response variable under model 4 (the temporal-spatial heterogeneity model). The greater the parameter estimate the larger the comparative influence on the response variable. Black bars represent site scale variables, grey bars landscape scale and white bars time series variables.

We also found that model 4 (the temporal-spatial heterogeneity model) was always ranked the highest and model 1 (the local habitat model) the lowest based on Akaike weight performance rankings (Table 4). The order of rankings for model 2 (spatial heterogeneity) and model 3 (temporal rainfall variability) varied depending on species, but there was very little difference in Akaike weights between models 2 and 3, and model 1 (Table 4). While the inclusion of landscape-scale heterogeneity improved the performance of all models, the direction of response for each landscape variable varied among species, although most species responded positively to spatial mean ground vegetation cover (Figure 3).This result reflects the variability in the individual species behavioural traits (Table 1).

**Figure 4:**
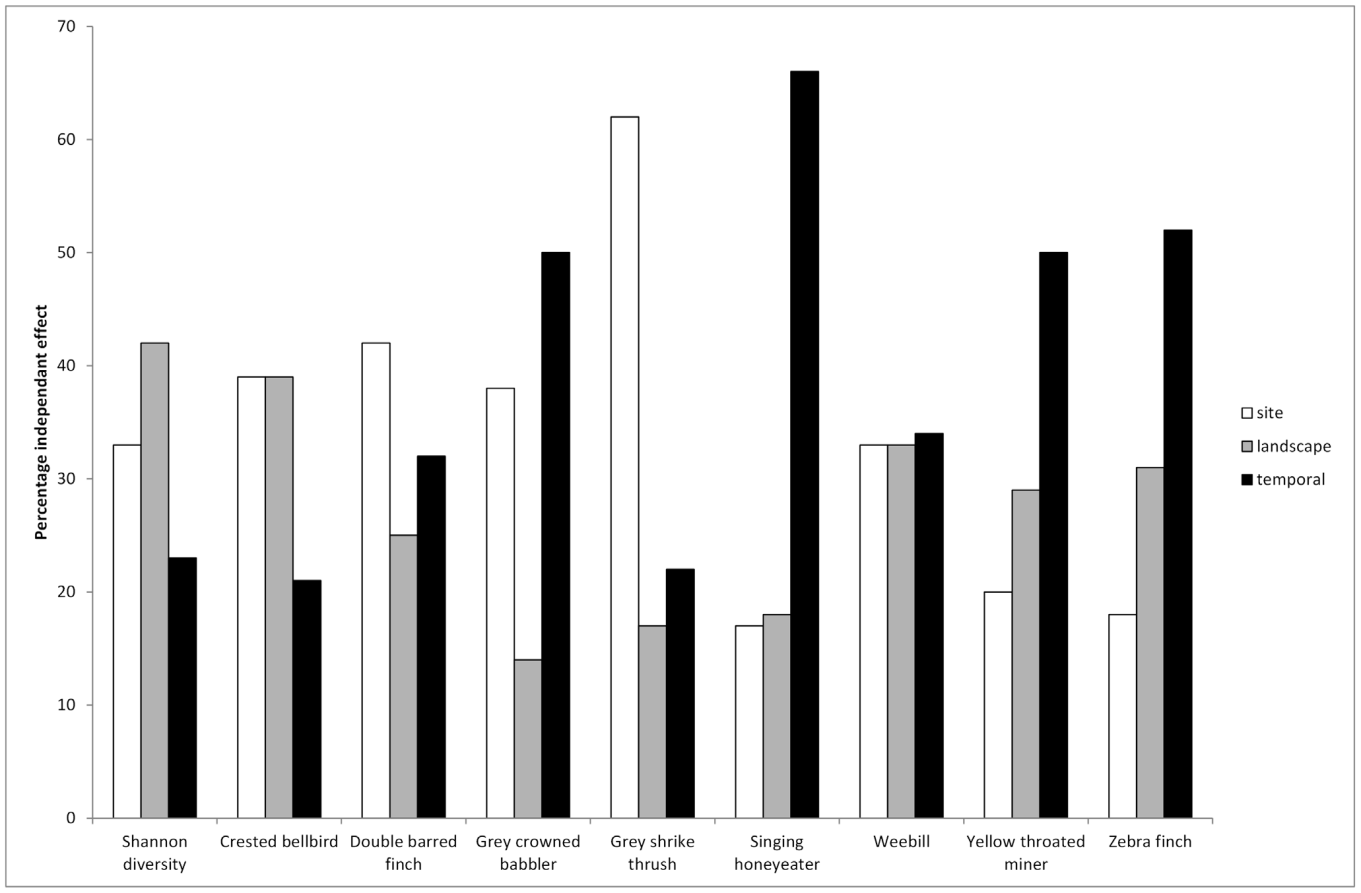
Total percentage independent effects resulting from hierarchical partitioning for model variables in each category for each species.

Hierarchical partitioning was performed on the full set of explanatory variables in the temporal and spatial variability model (model 4) since this model had the strongest support. The results revealed that temporal variables had particularly high independent effects for the yellow throated miner, grey crowned babbler, zebra finch and the singing honeyeater. Site-scale variables were particularly influential for the grey shrike thrush and the double barred finch. There was little differentiation between the site, landscape and temporal percentage independent effect for the weebill, crested bellbird and Shannon diversity for all birds (Figure 4).

## Discussion

This study has made an important contribution to disentangling the effects of landscape spatial and temporal heterogeneity on the diversity and abundance of fauna populations in highly dynamic environments such as Australia’s tropical savannas. We have taken advantage of multi-temporal archival Landsat Thematic Mapper imagery to quantify the influence of habitat variability on woodland birds at the appropriate spatial and temporal scales, thereby improving the predictive power of static species’ distribution models. Our results highlight the importance of spatio-temporal habitat dynamics in savanna landscapes and the importance of defining habitat on a species-specific basis [50], [51]. We found significant variability in the direction of response of individual species to both spatial and temporal variability in vegetation cover (ground and foliage projected cover). As such, the results of this study provide important support and information for more recent shifts in thinking regarding landscape-scale approaches to rangeland’s conservation management namely that: management for conservation of pattern and process should focus regimes to promote a shifting mosaic across large landscapes including highly variable and disturbed patches [52]; and complex landscape-scale effects on avian assemblage in mosaic environments are poorly understood and need more clear articulation via the use of a range of landscape metrics and tools [53].

Species diversity showed a positive response to both temporal and spatial mean ground cover and negative response to spatial and temporal variability in vegetation cover, suggesting a link between overall woodland bird diversity and reduced spatial variability in vegetation cover and higher average ground cover at the landscape-scale. Overall, landscape-scale and site-scale spatial variables had higher independent effects on species diversity than temporal variables. This pattern supports the concept that while seasonal variation in vegetation cover can drive changes in composition of the bird community, there is still a core bird assemblage that is strongly linked to the amount of habitat resources present in a landscape over the long-term [54]. Similar results have been found for the Australian arid zone by Pavey and Nano [25].

In savanna environments, regional heterogeneity in vegetation communities and cover is controlled largely by climate and soil characteristics, but at local to landscape scales, fire and grazing management practice (both natural and anthropogenic) can play a significant role [29]. Our results demonstrate that woodland bird species respond to spatial heterogeneity in vegetation cover at a finer spatial grain than that at which climatic variability can be measured. This has important implications for biodiversity conservation since vegetation heterogeneity within extensive tropical savanna ecosystems can be strongly influenced by management actions such as burning and grazing [29]. We have demonstrated that woodland bird species responses to this temporal variability are significant in a savanna landscape. Our results also suggest that the spatial dynamics of vegetation cover over the previous decades can have a significant influence on woodland bird diversity and abundance in savanna landscapes. The history of habitat heterogeneity at medium to long term can have important influence on species assemblages, and a recent examination of 10 year changes in woody vegetation cover using remote sensing in southern Africa identified change and impact on bird composition can be rapid [55].

At a species level, there was a variety of responses, predicated on the differences in the life history and ecology of each species. For the weebill, a small, ubiquitous and sedentary canopy dwelling species, there was no significant difference in the performance of the four models and it is seemingly resilient to land use change (e.g. fire, cattle grazing, tree thinning) [56], [57]. Conversely local scale habitat models were significant for species such as the grey shrike thrush and the double barred finch with strong association with dense patches of vegetation for breeding and shelter [58]. Temporal variability had the strongest independent effect on species such as the yellow throated miner, grey crowned babbler, zebra finch and the singing honeyeater; these species are highly mobile and move through landscapes according to changing climatic, resource and habitat conditions [58]. The yellow throated miner is disturbance-tolerant, and can colonize rapidly where there is habitat modification [59], [60], whereas the zebra finch and singing honeyeater migrate across landscapes in response to rainfall and resource pulses [6].

Globally, savanna landscapes are facing increasing and changing land use pressures, which will likely have serious implications for their fauna biodiversity [2]; [61]. Anthropogenic related grazing and fire disturbances are complex phenomena and interact over time periods of decades or longer [62]. Consequently, the capacity to distinguish between natural and anthropogenic impacts is an important challenge faced by savanna monitoring techniques [12], [63]. A recent review of alternative paradigms for rangelands conservation management suggested that the focus should shift to managing landscape heterogeneity and understanding the role of shifting and mosaic disturbance regimes caused by fire and grazing [52]. The relative effects of scale on the relationships between spatial pattern and avian species richness and composition are still considered not well known [53] but are required to assess and monitor global savanna ecosystems. Remotely sensed methods of monitoring the spatio-temporal variability in woody and herbaceous vegetation, such as those used in this study, offer a potentially useful and cost-effective approach to disentangle these complex relationships [13] [53] [64].

## Conclusions

This study advances our understanding of the relative importance of landscape spatial and temporal heterogeneity on fauna populations in highly dynamic environments such tropical savannas. It demonstrates that:

- Using remote sensing technology allows for combining spatial and temporal measures of habitat heterogeneity across several scales and significantly improves our ability to explain and understand savanna bird species dynamics and the complex relationship with landscape scale.
- While seasonal variation in vegetation cover can drive changes in composition of the bird community, core bird assemblages are strongly linked to the average amount of habitat resources present in the landscape and longer term (20 years) stability in vegetation cover.
- Species show individual responses to temporal and spatial changes in savanna landscapes, and as such habitat and landscape conservation goals must be both understood and defined on a species-specific basis.
- Temporal heterogeneity in vegetation cover at the local to landscape scale is shown to be of importance for explaining patterns of diversity and abundance in savanna birds and can have a greater influence than regional rainfall variability. This supports recent changes in thinking regarding rangeland ecology in that the management for landscape heterogeneity and mosaics of different disturbance regimes over longer time scales is more critical for conservation of native biota compared to more traditional utilitarian goals of short term sustainable, homogenous land management ideals.
